# Job vacancy at ECDC: Chief Scientist / Head of Unit Scientific Evidence and Communication

**DOI:** 10.2807/1560-7917.ES.2026.31.35.202609037

**Published:** 2026-09-03

**Authors:** Eurosurveillance editorial team

**Affiliations:** 1 European Centre for Disease Prevention and Control (ECDC), Stockholm, Sweden

The European Centre for Disease Prevention and Control (ECDC) invites applications for the following position:

**Chief Scientist / Head of Unit Scientific Evidence and Communication**
The application deadline expires on 30 September 2026.

For more information, please visit the ECDC recruitment platform: https://erecruitment.ecdc.europa.eu/en

